# Potential of ultra-high-resolution CT in detecting osseous changes of temporomandibular joint: experiences in temporomandibular disorders

**DOI:** 10.1186/s12903-023-03449-2

**Published:** 2023-10-09

**Authors:** Ning Zhang, Ruowei Tang, Pengfei Zhao, Ning Xu, Fanhao Meng, Zhen Wang, Tingting Zhang, Zhengyu Zhang, Hongxia Yin, Heyu Ding, Xiaoyu Qiu, Chihang Dai, Yan Huang, Zhenghan Yang, Xiaofeng Huang, Zhenchang Wang

**Affiliations:** 1 Department of Stomatology, Beijing Friendship Hospital, Capital Medical University, Beijing, China; 2 Department of Radiology, Beijing Friendship Hospital, Capital Medical University, Beijing, China

**Keywords:** Temporomandibular Joint Disorders, Tomography, X-Ray computed, Cone-Beam Computed Tomography, Diagnostic imaging

## Abstract

**Background:**

Osseous changes of the temporomandibular joint (TMJ) are related to the progression of temporomandibular disorders (TMD), and computed tomography (CT) plays a vital role in disease evaluation.

**Objective:**

The aims of this study were to evaluate the image quality and diagnostic value of ultra-high-resolution CT (U-HRCT) in TMD compared to cone-beam CT (CBCT).

**Methods:**

TMD patients who underwent both CBCT and U-HRCT between November 2021 and September 2022 were retrospectively included. Image quality scores were assigned for four osseous structures (the cortical and trabecular bones of the condyle, articular eminence, and glenoid fossa) by two independent observers from Score 1 (unacceptable) to Score 5 (excellent). Diagnostic classification of TMD was categorized as follows: Class A (no evident lesion), Class B (indeterminate condition) and Class C (definitive lesion). Image quality scores and diagnostic classifications were compared between CBCT and U-HRCT. The Cohen’s Kappa test, Wilcoxon signed-rank test, Chi-square test and Fisher’s exact test were conducted for statistical analysis.

**Results:**

Thirty TMD patients (median age, 30 years; interquartile range, 26–43 years; 25 females) with 60 TMJs were enrolled. Image quality scores were higher for U-HRCT than for CBCT by both observers (all *P*s < 0.001). Definitive diagnoses (Class A and C) were achieved in more cases with U-HRCT than with CBCT (93.3% *vs.* 65.0%, Fisher’s exact value = 7.959, *P* = 0.012). Among the 21 cases which were ambiguously diagnosed (Class B) by CBCT, definitive diagnosis was achieved for 17 cases (81.0%) using U-HRCT.

**Conclusions:**

U-HRCT can identify osseous changes in TMD, providing improved image quality and a more definitive diagnosis, which makes it a feasible diagnostic imaging method for TMD.

## Key Results

In a retrospective study of 30 participants (60 temporomandibular joints, TMJ), ultra-high-resolution CT (U-HRCT) showed higher image quality scores for four osseous structures of the TMJ by two independent observers, as compared to cone-beam CT (CBCT) (all *P*s < 0.001).

U-HRCT achieved more definitive diagnoses than CBCT (93.3% *vs.* 65.0%, Fisher’s exact value = 7.959, *P* = 0.012), and clarified diagnosis in 81.0% (17/21) cases which were ambiguously diagnosed by CBCT.

## Introduction

Temporomandibular disorders (TMD) are a series of multifactorial disorders presenting as craniofacial pain, limited mandibular motion, and joint sounds involving the temporomandibular joint (TMJ), masticatory muscles, and musculoskeletal structures [1, 2]. The cause of the disease remains unclear and is possibly related to trauma, severe pain stimuli, parafunctional activities, psychological elements, and genetics [3, 4]. The Diagnostic Criteria for Temporomandibular Disorders (DC/TMD), the most well-recognized classification system for TMD, have shown excellent reliability and validity [5–8]. DC/TMD have two assessment components: axis I and axis II, and the former classifies TMD into three groups: group I (muscular disorders), group II (disc displacement), and group III (arthralgia) [5].Although some patients present with short-term, mild, and self-limiting symptoms, others may experience chronic, persistent symptoms accompanied with physical, behavioral, and psychosocial alterations [1, 9].

The morphology of the osseous structures plays an important role in jaw movement, such as chewing, swallowing, and phonation [10]. Osseous changes of the TMJ are closely related to clinical symptoms of TMD and are important indicators for disease diagnosis, staging, and treatment strategy. As repair and regeneration of the condyle are possible in early-stage TMD, identification of the osseous alterations is vital [11]. Osseous changes that can be visualized by computed tomography (CT), such as subchondral cyst, erosion, generalized subchondral sclerosis and osteophyte, are indicative of TMD [12].

One of the most important advancements for TMD diagnosis is in the development of imaging techniques [1]. Among these imaging methods, cone-beam CT (CBCT) is regarded as the method of choice for evaluating bony morphology [13, 14]. However, no consensus has been reached on the sensitivity and specificity of CBCT for detecting bony changes of the TMJ [14–16], and there is a report stating that CBCT may not be suitable as a screening method for TMD [17]. In addition, the interobserver agreement for the condyle morphology classification by CBCT was low, which may be attributed to the obscure depiction of the osseous structures and to the operator-dependent selection of image slices [18]. Therefore, accurate diagnosis is dependent on images with improved visualization, particularly in cases with indeterminate diagnosis on CBCT.

Recently, ultra-high-resolution CT (U-HRCT), a newly-developed device, has been introduced as a reliable method for imaging delicate structures of the temporal bone. Its application on cadaveric specimens, healthy participants, and patients with temporal bone diseases has been validated in previous studies [19–21]. However, the application on the TMJ has not been reported yet.

Since better diagnostic method advances the understanding of disease prevalence, incidence, and pathological progression [7], the aims of the present study were two-fold: (1) to evaluate the image quality of the TMJ on U-HRCT with reference to CBCT, and (2) to assess the diagnostic value of U-HRCT in terms of detecting osseous changes in TMD patients.

## Materials and methods

### Eligible participants

**Fig. 1:**
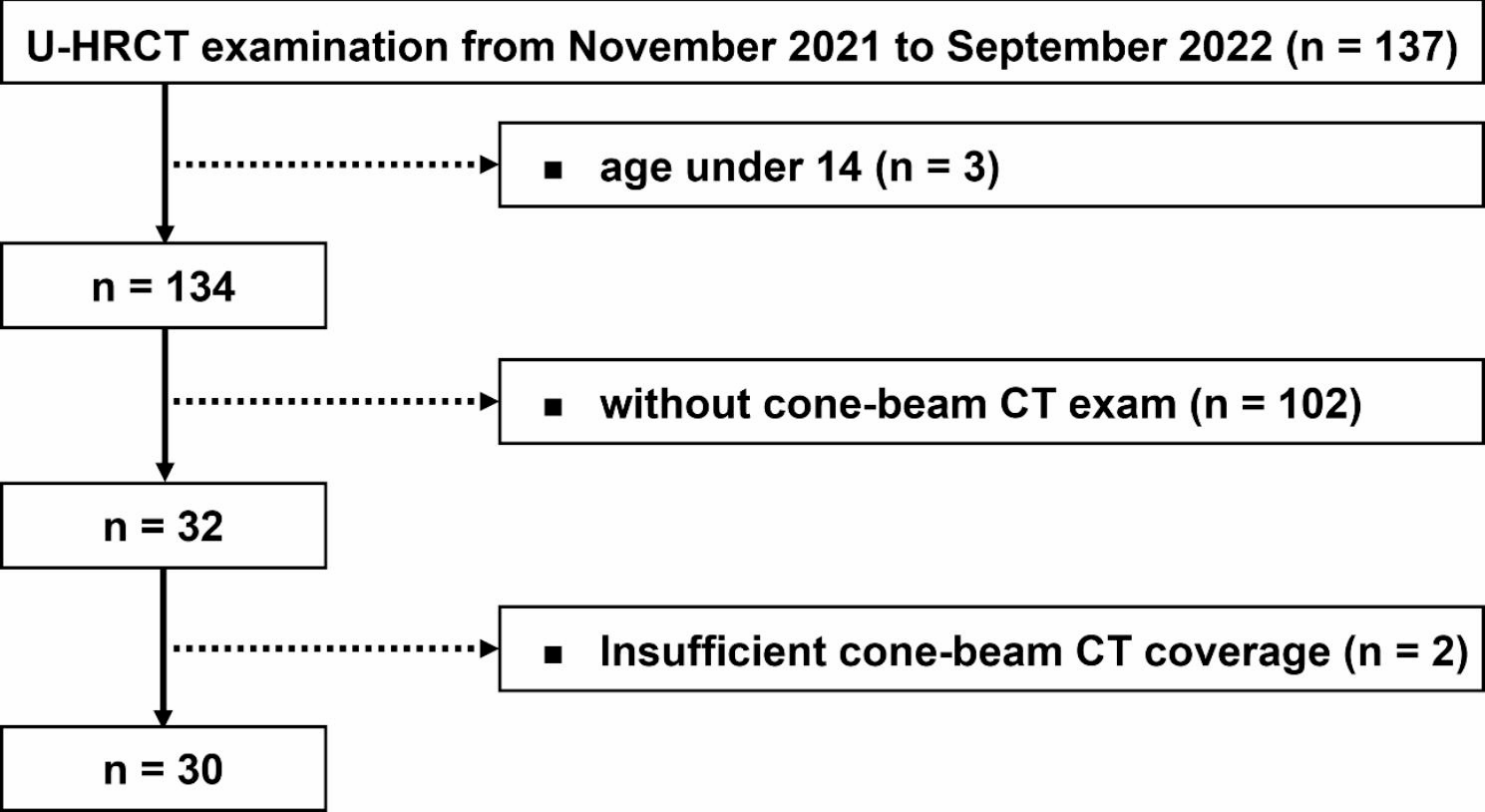
Flowchart of the study design

This retrospective study was approved (No. 2022-P2-366-01) and the informed consent was waived by the Bioethics Committee of Beijing Friendship Hospital, Capital Medical University, owing to its retrospective design.The inclusion criteria were TMD patients: (1) who were suspected as group II or III according to DC/TMD and presented with clinical symptoms as TMJ clicking, noise, limited mouth opening and pain and (2) who underwent U-HRCT examination between November 2021 and September 2022 (n = 137). The exclusion criteria were as follows: (1) age under 14 years (n = 3), (2) patients who did not undergo CBCT examination (n = 102), and (3) patients with insufficient CBCT image coverage (n = 2). Finally, 30 patients with 60 TMJs were included for further analysis (Fig. 1). No patient underwent both U-HRCT and CBCT examinations for the purpose of this study.

### Imaging acquisition

#### CBCT protocol

TMD patients were examined with a CBCT scanner (NewTom 5G Version FP, QR S. r. l, Italy) at 110 kVp, 5 mA. The field-of-view was 18 cm × 16 cm, with a voxel size of 0.3 mm and exposure time of 3.6 s.

#### U-HRCT protocol

The TMJs were scanned unilaterally with the U-HRCT scanner (Ultra3D, LargeV, Beijing) at 100-110 kVp and 120-180 mAs with a field-of-view of 65 mm. The slice thickness and interval were both set at 0.1 mm. Consequently, isotropic axial images that could be reformatted from any desired direction were acquired. The exposure time was 20 s.

### Imaging analysis

#### Image quality assessment

Image quality was independently evaluated by an oral and maxillofacial surgeon (N. Z., 12-year experience in imaging reading) and a radiologist (R.T., 7-year experience), both of whom were blinded to clinical data of the patients. The CBCT and U-HRCT images were reviewed using the QR-NNT Viewer (ver. 5.6.0, Quantitative Radiology, Verona, Italy) and RadiAnt DICOM Viewer (ver. 2021.2, Medixant, Poznan, Poland), respectively. Both CBCT and U-HRCT images were viewed in a dimly lit, quiet room. The observers were allowed to adjust the zoom, brightness, and contrast as necessary.

**Fig. 2:**
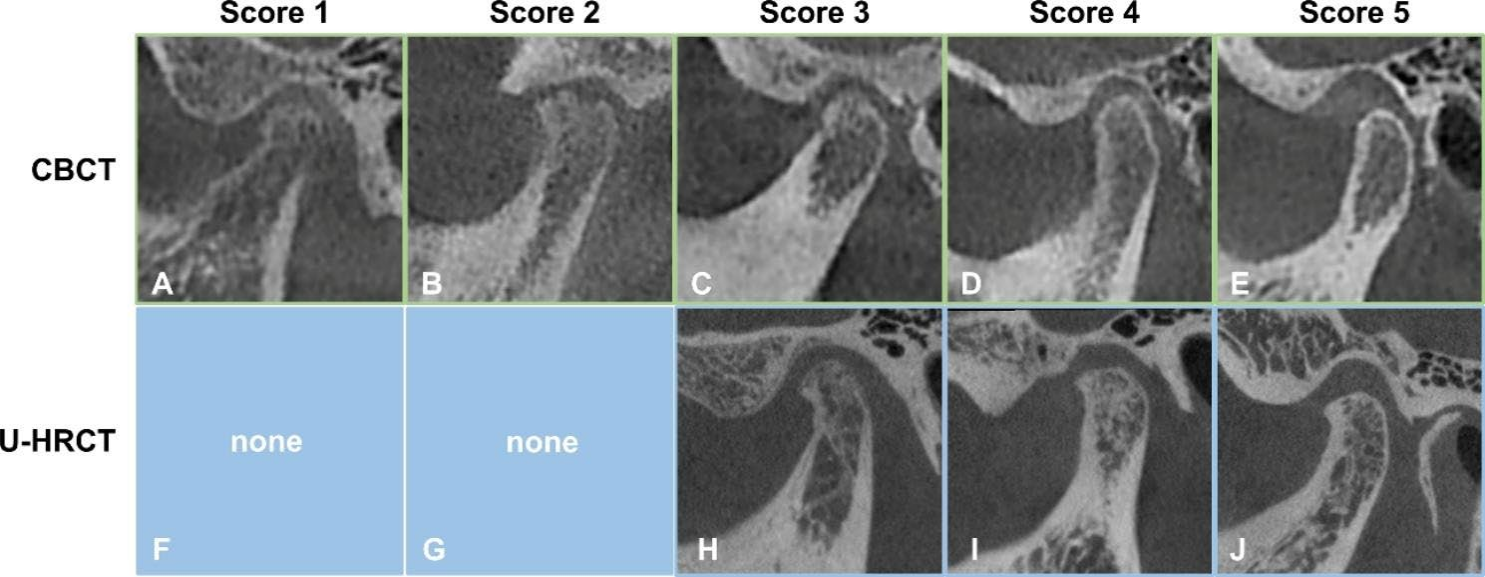
Image quality scores of CBCT and U-HRCT. Score 1 = unacceptable, nondiagnostic image quality (A); Score 2 = poor image quality, limited diagnostic value (B); Score 3 = fair, diagnostic image quality with mild artifacts or noise (C and H); Score 4 = good, diagnostic image quality to detect osseous changes (D and I); and Score 5 = excellent diagnostic image quality without artifacts or noise, optimal to make a diagnosis (E and J). Note that no cases are scored 1–2 for U-HRCT (F and G)

Image quality of four osseous structures of the TMJ, namely the cortical bone of the condyle, trabecular bone of the condyle, articular eminence, and glenoid fossa, was rated using a modified 5-point Likert scale as follows [22] (Fig. 2):

Score 1 = unacceptable, nondiagnostic image quality because of severe artifacts or excessive noise;

Score 2 = poor image quality, limited diagnostic value, the structure was visible but difficult to analyze due to moderate artifacts or noise;

Score 3 = fair, diagnostic image quality with mild artifacts or noise;

Score 4 = good, diagnostic image quality to detect osseous changes;

Score 5 = excellent diagnostic image quality without artifacts or noise, optimal to make a diagnosis.

### Diagnostic value of U-HRCT for TMD

The two observers first made a primary diagnosis on the basis of radiographic features on U-HRCT and CBCT independently; these diagnoses were irrespective of the image quality to simulate the real-world clinical setting. For cases with any disagreement between the two observers, a third senior observer (X. H., 23 years of experience) was introduced to interpret images and make the final diagnosis.

**Fig. 3:**
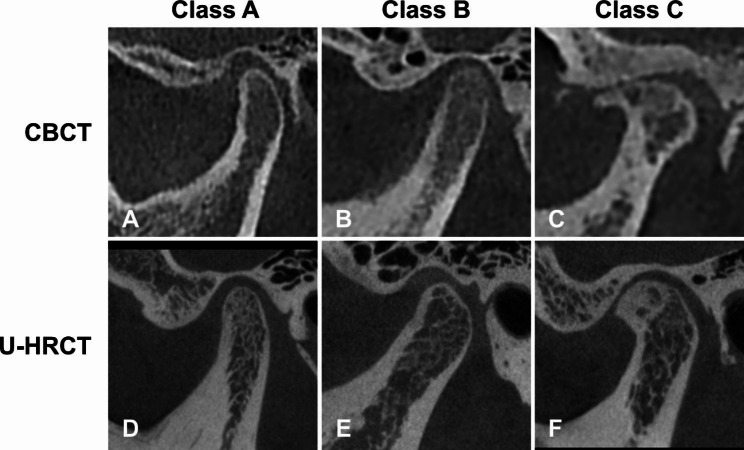
Diagnostic classification for TMD using CBCT and U-HRCT. Class A = no evident lesion (A and D), Class C = definitive lesion, including generalized subchondral sclerosis, osteophyte, erosion of the condyle, subchondral cyst and condylar flattening (C and F), and Class B = ambiguous condition where a case could not be diagnosed as Class A or C (B and E)

The diagnosis of TMD was categorized into the following classifications: Class A = no evident lesion; Class C = definitive lesion, including generalized subchondral sclerosis, osteophyte, erosion of the condyle, subchondral cyst and condylar flattening [12]; and Class B = ambiguous condition where a case could not be diagnosed as Class A or C (Fig. 3). Cases graded as Class A and C were considered with definitive diagnoses, whereas those graded as Class B were considered with indeterminate diagnoses.

### Statistical analysis

Statistical analyses were performed using SPSS (ver. 26.0, Armonk, NY, IBM Corp) and GraphPad Prism 7 (GraphPad Software, La Jolla, CA, USA). Interobserver agreement in terms of the image quality score was tested using the Cohen’s Kappa test, and the strength of the agreement was rated as follows: slight 0.00–0.20, fair 0.21–0.40, moderate 0.41–0.60, good 0.61–0.80, and excellent 0.81–1.00. Image quality scores were expressed as the median (interquartile range, IQR) and were compared between the two CT modalities using the Wilcoxon signed-rank test. The percentages of cases with definitive diagnoses (Class A and C) made by U-HRCT and CBCT were compared using Chi-square test or Fisher’s exact test. *P* values less than 0.05 were considered statistically significant.

## Results

### Demographic data of participants

A total of 30 participants (60 TMJs) with unilateral or bilateral TMD were included in this study (median age, 30 years; IQR, 26–43 years; 25 women). Among these TMJs, 37 (61.7%) were clinically diagnosed as TMD with at least one of the following symptoms: TMJ clicking, noise, mouth opening limitation, and pain. In the affected TMJs, 23/37 (62.2%) and 14/37 (37.8%) TMJs were clinically categorized as group II and III, respectively, according to DC/TMD. Other demographic characteristics are summarized in Table 1.

**Table 1:** Baseline demographic data of participants

Characteristic	Value
No. of participants	30
Median age (y, IQR)	30 (26–43)
Sex, n (%)	
F	25 (83.3)
M	5 (16.7)
Unaffected TMJ, n (%)	23 (38.3)
Affected TMJ, n (%)	37 (61.7)
Side	
Right	20 (54.1)
Left	17 (45.9)
DC/TMD classification, n (%)	
Group II	23 (62.2)
Group III	14 (37.8)
Onset duration (month)	19.2 (0.25–120)
Clinical manifestation, n (%) ^*^	
TMJ clicking	10 (16.9)
TMJ noise	13 (22.0)
Mouth opening limitation	15 (25.4)
TMJ pain	21 (35.6)

IQR, interquartile range; TMJ, temporomandibular joint; DC/TMD, Diagnostic Criteria for Temporomandibular Disorders

^*^The affected TMJs may present with one, two or more clinical manifestations, thus the sum of this entry is 59 TMJs.

### Interobserver agreement

For U-HRCT, the observers showed good-to-excellent agreement (Cohen’s Kappa coefficients range: 0.78–0.88), with the lowest value for the trabecular bone and the highest for the articular eminence. For CBCT, the Cohen’s Kappa coefficients were moderate-to-good (0.58–0.77), with the lowest for the trabecular bone and the highest for the articular eminence (Table 2).

**Table 2:** Image quality score on U-HRCT and CBCT by the two observers

Image quality score, median (interquartile range)	Observer 1				Observer 2				Cohen’s Kappa coefficient (95% confidence interval)	
	U-HRCT	CBCT	*Z* value	*P* value^*^	U-HRCT	CBCT	*Z* value	*P* value^*^	U-HRCT	CBCT
Condyle										
Cortical bone	4 (4–5)	3 (3–4)	-6.317	< 0.001	4 (4–5)	3 (3–4)	-6.367	< 0.001	0.83 (0.69–0.97)	0.64 (0.47–0.81)
Trabecular bone	4 (4–5)	3 (3–3)	-6.553	< 0.001	4 (4–5)	3 (2–3)	-6.701	< 0.001	0.78 (0.64–0.92)	0.58 (0.40–0.76)
Articular eminence	5 (5–5)	4 (3–4)	-6.836	< 0.001	5 (5–5)	4 (3–4)	-6.859	< 0.001	0.88 (0.65–1.11)	0.77 (0.61–0.93)
Glenoid fossa	5 (5–5)	3 (3–4)	-6.828	< 0.001	5 (5–5)	4 (3–4)	-6.816	< 0.001	0.82 (0.57–1.07)	0.74 (0.59–0.89)

### Comparison of image quality score

For observer 1, image quality scores of the cortical bone of the condyle, trabecular bone of the condyle, articular eminence, and glenoid fossa on CBCT images were 3 (3–4), 3 (3–3), 4 (3–4), and 3 (3–4), respectively. The corresponding scores on U-HRCT images were 4 (4–5), 4 (4–5), 5 (5–5), and 5 (5–5), respectively. For observer 2, the image quality scores for the same regions on CBCT images were 3 (3–4), 3 (2–3), 4 (3–4), and 4 (3–4), respectively. The corresponding scores using U-HRCT images were 4 (4–5), 4 (4–5), 5 (5–5), and 5 (5–5), respectively. The image quality scores were higher for U-HRCT than for CBCT by both observers (all *P*s < 0.001, Table 2). An overview of image quality scores by the two observers is shown in Table 3, showing that image quality scores of U-HRCT were not inferior to those of CBCT for any case.

**Table 3:** Comparison of image quality score on U-HRCT and CBCT

Structures, n	Observer 1			Observer 2		
	CBCT+	=	U-HRCT+	CBCT+	=	U-HRCT+
Condyle						
Cortical bone	0	10	50	0	10	50
Trabecular bone	0	6	54	0	3	57
Articular eminence	0	4	56	0	3	57
Glenoid fossa	0	2	58	0	3	57

Four osseous structures were analyzed according to a 5-point Likert scale in 60 temporomandibular joints in U-HRCT and CBCT by observers 1 and 2. “CBCT +” represents CBCT is given higher score than U-HRCT. “=” represents equal scores for CBCT and U-HRCT. “U-HRCT +” represents U-HRCT is given higher score than CBCT

### Diagnostic classification of TMD

For the diagnostic classification of TMD, 13.3% (8/60), 35% (21/60), 51.7% (31/60) cases in CBCT and 21.7% (13/60), 6.7% (4/60) and 71.7% (43/60) in U-HRCT were categorized as Class A, B and C, respectively. Using CBCT, definitive (Class A and C) and indeterminate (Class B) diagnoses were made in 65.0% (39/60) and 35.0% (21/60) cases, respectively. Meanwhile, definitive diagnoses were achieved in 93.3% (56/60) cases, and the remaining 6.7% (4/60) cases were ambiguously diagnosed by U-HRCT. The percentage of definitive diagnoses achieved with U-HRCT is much higher than that with CBCT (Fisher’s exact value = 7.959, *P* = 0.012, Table 4).

**Table 4:** Cases with discrepant diagnosis by U-HRCT and CBCT

CBCT	U-HRCT	
	Definitive diagnosis (n = 56)	Indeterminate diagnosis (n = 4)
Definitive diagnosis (n = 39)	39	0
Indeterminate diagnosis (n = 21)	17	4
Fisher’s exact test	7.959	
*P* value	0.012	

**Fig. 4:**
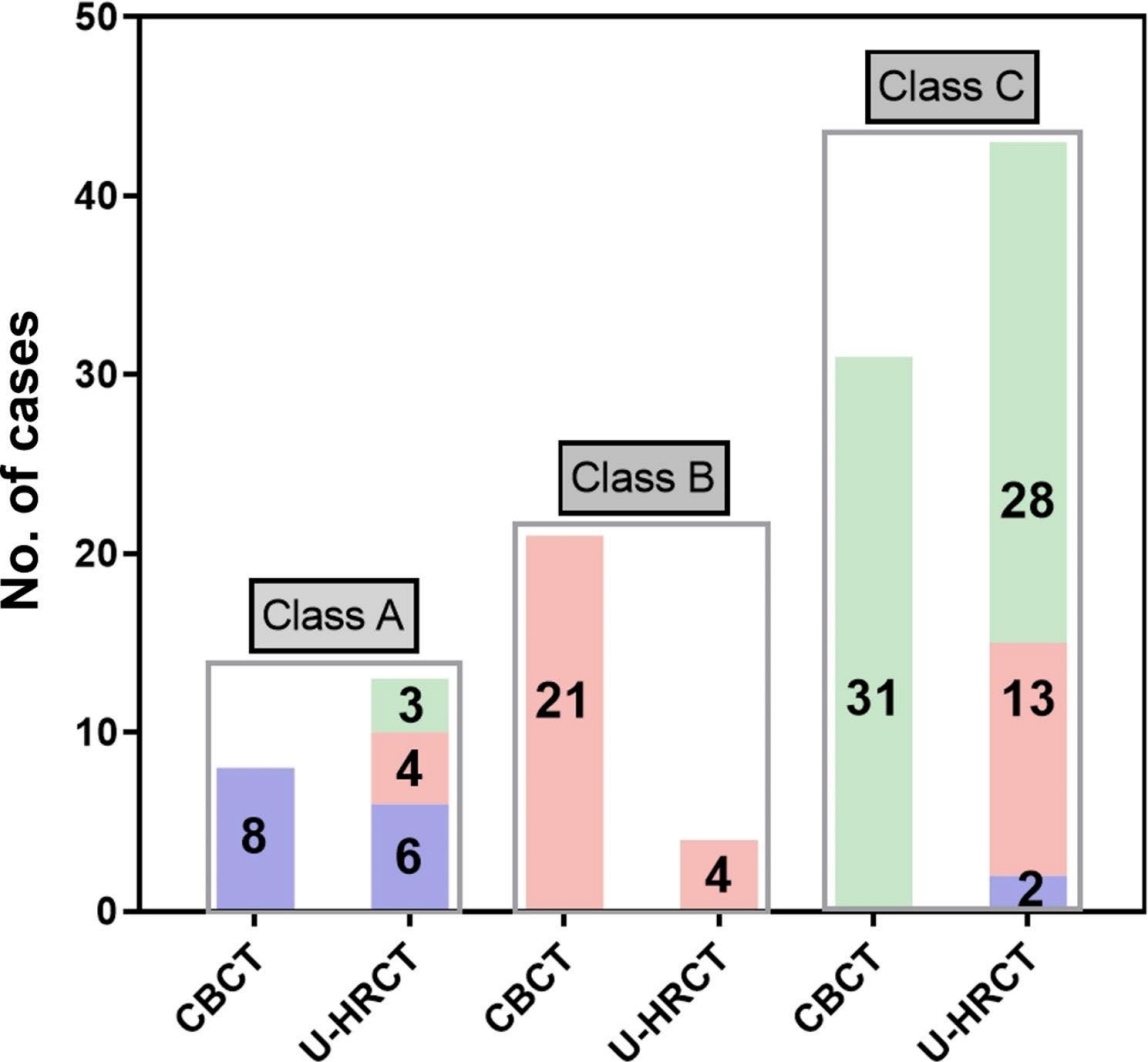
Distribution of the diagnostic classification of 60 TMJs. Column colors are indicative of a diagnosis by CBCT as follows: purple for Class A, pink for Class B, and green for Class C. For Class A by U-HRCT, 6 cases (purple) are consistent with the diagnosis on CBCT, 3 cases (green) are revised from a diagnosis of Class C on CBCT, and 4 cases (pink) are revised from a diagnosis of Class B on CBCT. For a diagnosis of Class C on U-HRCT, 28 cases (green) are consistent with the diagnosis on CBCT, 13 cases (pink) are revised from a diagnosis of Class B on CBCT, and 2 cases (purple) are revised from a diagnosis of Class A on CBCT.

More specifically, among the 39 cases with a definitive diagnosis on CBCT, 34 (87.2%) cases showed consistent results on U-HRCT. Diagnoses of the remaining 5 (12.8%) cases were revised as follows: 2 cases from Class A (CBCT) to Class C (U-HRCT) and the other 3 cases from Class C (CBCT) to Class A (U-HRCT; Fig. 4).

**Fig. 5:**
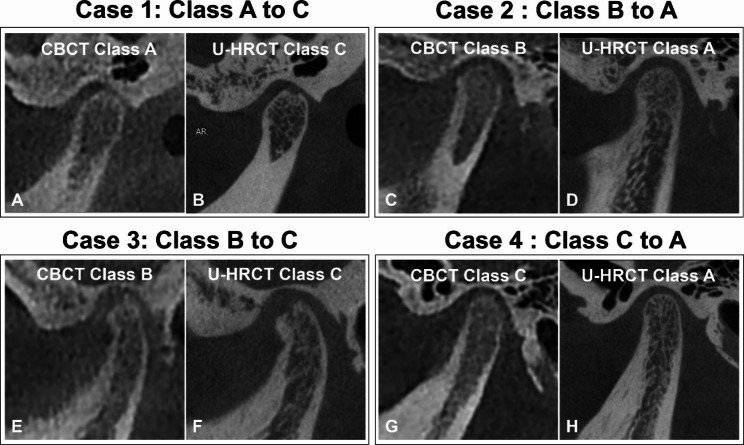
Discrepant diagnoses by CBCT and U-HRCT. (A-B) Left TMJ of a 41-year-old woman who is assigned Class A (continuous cortical bone of the condyle without erosion) on CBCT but Class C (bony erosion on the roof of the condyle) on U-HRCT. (C-D) Right TMJ of a 16-year-old man who is assigned Class B (suspicious discontinuity on the posterior border of the condyle) on CBCT but Class A (no evident bony changes) on U-HRCT. (E-F) Left TMJ of a 20-year-old woman who is assigned Class B (suspicious discontinuity on the posterior border of the condyle and osteophyte on the roof) on CBCT but Class C (bony erosion and osteophyte of the condyle) on U-HRCT. (G-H) Right TMJ of a 16-year-old woman who is assigned Class C (suspicious bony discontinuity on the posterior border of the condyle) on CBCT but Class A (no evident bony changes) on U-HRCT.

Among cases with indeterminate diagnoses on CBCT, 81.0% (17/21) achieved definitive diagnoses using U-HRCT, and 19.0% (4/21) still had an indeterminate diagnosis (Fig. 4). In these cases, 13 were revised to Class C (U-HRCT) with depiction of the following radiographic findings: subchondral sclerosis (11/13, 84.6%), cystic change (5/13, 38.5%), condylar flattening (9/13, 69.2%), erosion (7/13, 53.8%), and osteophyte (10/13, 76.9%). In the 4 cases revised to Class A on U-HRCT, CBCT showed obscure, discontinuous bony cortex, whereas U-HRCT showed continuous, smooth bony cortex (Fig. 5). The remaining 4 cases were still undiagnosed as their imaging showed only local condylar flattening and were thus assigned as Class B by U-HRCT (Fig. 4).

## Discussion

In this study, U-HRCT was introduced to image the TMJ in patients with TMD. Using a 5-point Likert scale, U-HRCT showed significantly higher image quality scores for displaying osseous structures of TMJ (the cortical bone of the condyle, trabecular bone of the condyle, articular eminence and glenoid fossa) compared to CBCT. The interobserver agreement was good-to-excellent (Cohen’s Kappa coefficient: 0.78–0.88). Significantly more cases were definitively diagnosed using U-HRCT than using CBCT (93.3% *vs. *65.0%, *P* = 0.012). In particular, U-HRCT achieved a definitive diagnosis in 81.0% (17/21) of the cases with indeterminate diagnoses by CBCT.

CBCT has shown comparable or superior reliability to conventional spiral CT [16]; however, the heterogeneity on its sensitivity and specificity for TMD should be addressed. Several studies have reported low sensitivity (0.03–0.67) of CBCT in diagnosis of TMD [14, 16]. In addition, the interobserver agreement for the condylar morphology classification using CBCT was found to be low (Kappa coefficient = 0.181–0.265) and the authors concluded that CBCT was not suitable for shape classification [18]. Based on these studies, it is reasonable to postulate that CBCT may not be reliable and effective for identifying osseous changes and thus, may not be a suitable screening method for TMD [17].

U-HRCT, a newly-developed device with 0.1-mm spatial resolution, can provide diagnostic details through improved image quality. It has good image quality for delicate temporal bone structures and allows effective identification of normal and diseased conditions of the temporal bone; therefore, it has been used in the field of otolaryngology [19–21]. Overall, U-HRCT has shown good-to-excellent interobserver agreement [19], which is consistent with the findings of the present study, implying that it is a reliable diagnostic imaging method. These studies indicate that the application of U-HRCT in TMD patients is possible.

In our study, we assessed the diagnostic value for TMD by dividing the cases into three classifications: Class A, B, and C. The diagnostic value was considered higher when more cases were identified as having a definitive diagnosis (Class A or C). As illustrated in the results, significantly more cases reached definitive diagnoses with U-HRCT than with CBCT (93.3% *vs.* 65.0%, *P* = 0.012). Therefore, we concluded that with improved image quality, U-HRCT made it easier for the observers to make a confident diagnosis with reduced possibility of diagnosis discrepancy, which is particularly vital in the clinical setting.

Specifically, the diagnostic value of U-HRCT lies in its ability to make a definitive diagnosis in cases with ambiguous diagnoses by CBCT. We found that U-HRCT achieved definitive diagnoses in 81.0% (17/21) of cases with indeterminate diagnoses on CBCT (4 cases revised to Class A and 13 cases to Class C). U-HRCT could reach a definitive diagnosis because of the more detailed radiographic findings of TMJ. Notably, no agreement has been reached on whether flattening of the condyle was a pathological condition [23–25]. Therefore, we classified flattening only as an indeterminant sign for TMD on U-HRCT. Therefore, for the 4 cases classified as Class B on U-HRCT, the diagnosis was indeterminant not because of poor image quality but for the presence of only condylar flattening. The diagnostic ability to make an explicit diagnosis of TMD implied that U-HRCT had a lower risk of misdiagnosis. Therefore, U-HRCT could help diagnose TMD by identifying osseous changes, which could potentially benefit early-stage diagnosis, disease staging, and choice of treatment strategy.

The clinical implications of the current study lied in two aspects. First,owing to the good image quality, U-HRCT could identify early-stage osseous changes, which may benefit accurate diagnosis and treatment selection. Indeterminate diagnosis by CBCT could be clarified using U-HRCT, and radiographic findings of U-HRCT could revise the diagnosis made by CBCT. Second, we found that more cases were assigned Class C (evident lesions) by U-HRCT than assigned to group III by DC/TMD (43 cases *vs.* 14 cases). U-HRCT may detect more cases with bony changes compared with DC/TMD, since the sensitivity for degenerative disease was only 0.55 by DC/TMD without imaging examination [5]. The reason may be the inconsistency between the clinical manifestation and osseous changes, that is, evident bony changes may be found in patients with relatively mild symptoms. 

This study has several limitations that need to be acknowledged. First, although we included a cohort of TMD patients of group II (disc displacement) and group III (arthralgia), imaging features of different stages of TMD were not further compared. Nevertheless, U-HRCT consistently showed superior image quality and diagnostic value in the study cohorts of group II and group III patients. Second, we did not categorize patients into different age groups. As the cortical bone of the condyle is not fully established until the age of 21–22 years [26], the inclusion of 5 patients (10 TMJs) aged 15–22 years in this study may lead to overestimation of disease presence. Third, the correlation between imaging appearance of TMD and clinical manifestations, including long-term follow-up, was not discussed in this study, and this will be a topic of interest in a future study. Last, given that U-HRCT is a newly-developed device, its cost-effectiveness should be evaluated for further exploration.

In conclusion, U-HRCT provided higher image quality scores than CBCT with good-to-excellent interobserver agreement. U-HRCT achieved definitive diagnosis in 81.0% cases that were ambiguously diagnosed by CBCT, and revised diagnoses of 12.8% cases by providing detailed radiographic findings. Therefore, it may be a feasible diagnostic imaging method to identify osseous changes of TMD, thus helping clinicians reach an accurate diagnosis and make appropriate treatment decisions.

## Data Availability

The datasets used and/or analyzed during the current study are available from the corresponding author on reasonable request.

## Abbreviations

TMD: Temporomandibular disorders
TMJ: Temporomandibular joint
DC/TMD: Diagnostic Criteria for Temporomandibular Disorders
CT: Computed tomography
CBCT: Cone-beam CT
U-HRCT: Ultra-high-resolution CT
IQR: Interquartile range
